# Editorial: CAR T-cells: novel therapeutic approaches in the new era of cancer immunotherapy

**DOI:** 10.3389/fmmed.2023.1239013

**Published:** 2023-06-27

**Authors:** Alice Turdo, Costanza Maria Cristiani, Niels Schaft

**Affiliations:** ^1^ Department of Health Promotion, Mother and Child Care, Internal Medicine and Medical Specialties (PROMISE), University of Palermo, Palermo, Italy; ^2^ Department of Medical and Surgical Sciences, Neuorscience Research Center, “Magna Graecia” University of Catanzaro, Catanzaro, Italy; ^3^ Department of Dermatology, Friedrich-Alexander-Universität Erlangen-Nürnberg, Universitätsklinikum Erlangen, Erlangen, Germany; ^4^ Comprehensive Cancer Center Erlangen European Metropolitan Area of Nuremberg (CCC ER-EMN), Erlangen, Germany; ^5^ Deutsches Zentrum Immuntherapie (DZI), Erlangen, Germany

**Keywords:** CAR T-cell, cancer, immunotherapy, side effects, tumor microenvironment

## 1 Introduction

**FIGURE 1:**
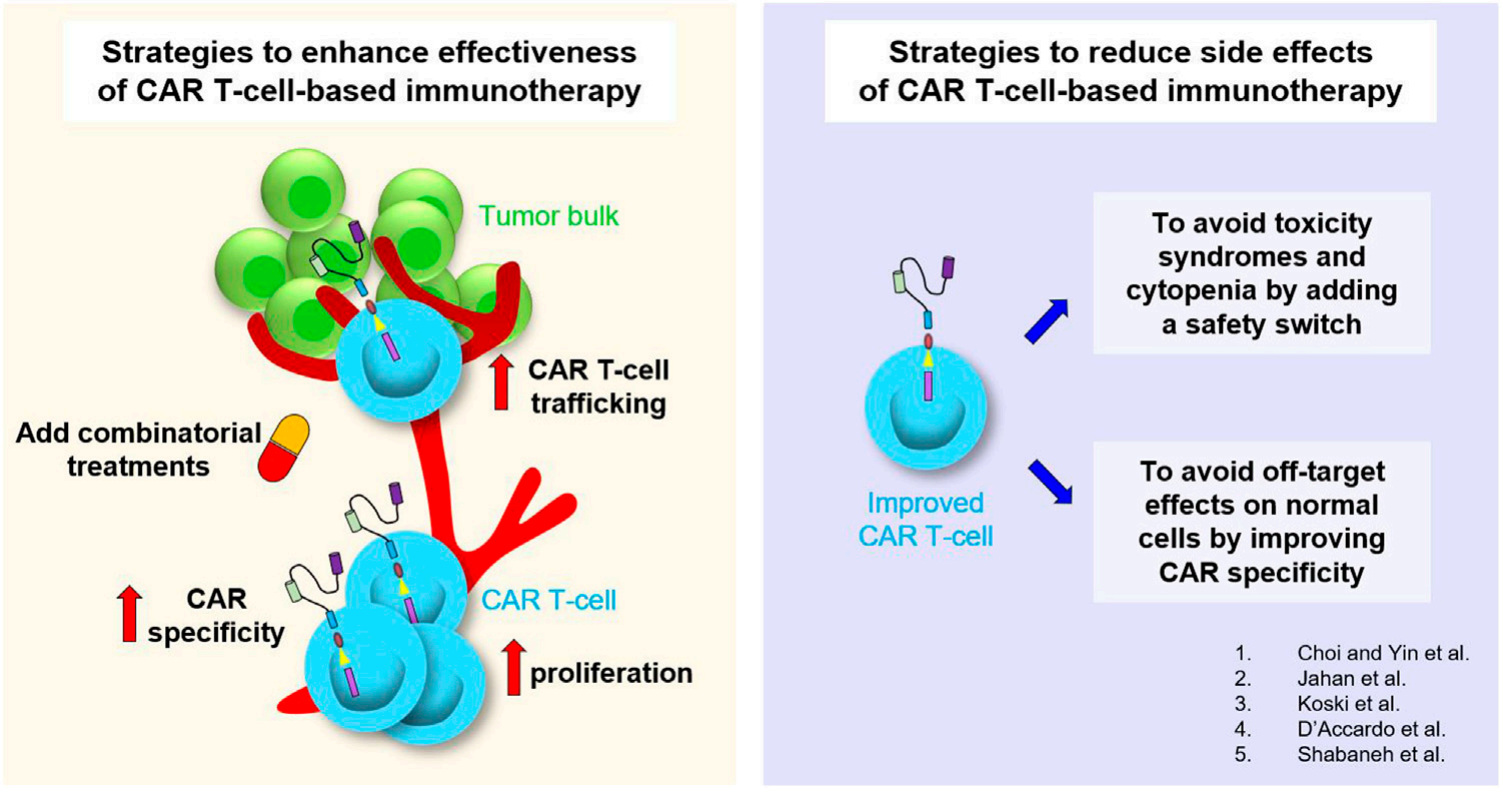
A Comprehensive overview of the studies published in this Research Topic.

Immunotherapy has emerged as one of the most effective treatments capable of overcoming tumor resistance mechanisms due to its ability to modulate the patient’s immune response against cancer. Personalized anti-tumor therapy based on T cells engineered to express a cancer-specific chimeric antigen receptor (CAR) acts directly on the immune system of patients. Specifically, this therapy enhances the recognition of cancer cells by T lymphocytes, thus promoting their elimination. In this Research Topic several aspects of CAR T-cell therapy, with particular emphasis on novel findings aimed at ameliorating the effectiveness of CAR T-cell-based immunotherapy and reducing side effects, are described (Figure 1).

## 2 Enhancing the effectiveness of CAR T-cell-based immunotherapy

Tremendous clinical regressions have been achieved using CAR T-cells against a variety of cancers, both in numerous preclinical studies and in several clinical trials, most notably against leukemia, lymphoma, and multiple myeloma, resulting in an FDA- and EMA-approval of these CAR T-cell therapies. Especially in solid tumors, the effectivity of CAR T-cell therapy is hampered by several factors, such as the immunosuppressive microenvironment, T-cell exhaustion, limited extravasation out of the blood vessel, or ineffective trafficking of CAR T-cells to tumor sites. Therefore, new strategies are needed to enhance the effectiveness of CAR T-cell-based immunotherapy.

In the review by Choi and Yin, the authors give their insights on prospective approaches to enhance this therapy in glioblastoma (GBM). They describe how CAR constructs can be modified to enhance specificity towards, or overcome the heterogenicity of GBM, and to improve CAR T-cell trafficking to the tumor. Furthermore, several strategies are presented to engineer CAR T-cells with gene editing to overcome T-cell dysfunctions. Finally, approaches with additional chemical and external treatments to improve CAR T-cell therapy are suggested.

For *in-vitro* testing, CAR T-cells are usually generated from healthy donors’ primary T cells. This implies the need to isolate and expand T cells, often accompanied by poor reproducibility in production and effectiveness. To address these issues, in their original research paper, Jahan et al. employed the reporter Jurkat cell line JE6.1 as a standardized cell platform to assess CAR function. Starting from the CAR structure (FiCAR) developed by Koski et al., the authors generated several variants differing for spacer length, as well as for target specificity. *In vitro*, CD19-targeted FiCARs were able to induce immune synapse formation, degranulation, cytotoxicity, and intracellular signaling in Jurkat cells. Similar results were observed by transducing primary T cells. The most successful FiCAR could also be effectively engineered to target solid tumor antigens, thus demonstrating that Jurkat cells may represent a valuable standardized tool to generate CAR T-cells, as well as to quickly assess the CAR’s function.

The recognition of tumor-associated antigens (TAA) by CAR T-cells triggers cell-mediated immunity and cytokine release, causing cancer-cell elimination. Although this supposedly straightforward mechanism of action has been successful in proof-of-principle studies, the efficacy of CAR T-cells may vary according to diverse circumstances.

D’Accardo et al. reviewed that physical barriers in solid tumors may impair CAR T-cell trafficking to the tumor site and that an immunosuppressive tumor microenvironment (TME) could cause a scarce persistence of CAR T-cells. Additionally, an aberrant recognition of antigens, provoked by fluctuation in the levels of tumor antigens, and the refractoriness to the targeting of a single antigen, have been described as a major hurdle in CAR T-cell immunotherapy.

In order to improve treatment outcomes, major advances in the design of engineered cellular therapies have been conceptualized, including the optimization of lymphocyte metabolism and the choice of powerful target antigens expressed by the aggressive subpopulation of cancer stem cells responsible for the onset and progression of tumors.

## 3 Reducing side effects of CAR T-cell-based immunotherapy

Notwithstanding, CAR T-cells showed efficacy in liquid malignancies as well as in solid tumors, and meticulous descriptions of side effects and treatment failures have been highlighted in clinical studies.

As comprehensively reviewed by D’Accardo et al., limitations to the CAR-T therapy are ascribable to the onset of side effects including the syndrome related to the release of cytokines (CRS), the neurologic syndromes associated with immune cells (ICANS), and cytopenia. Furthermore, “off-target” effects may occur due to the unspecific killing of non-cancerous cells.

In this context, Shabaneh et al. developed a valid approach, based on antibody-dependent cellular cytotoxicity, to overcome side effects generated by CAR T-cell therapies. In this study, the expression on the CAR T-cell surface of an optimized peptide derived from the EGFR (EGFRopt) acted as a safety switch following targeting by cetuximab.

Similar to the already described safety switching systems, based on the expression of inducible caspase 9, the human thymidylate kinase, and the herpes simplex virus tyrosine kinase, the EGFRopt system could reverse serious toxicities due to the rapid elimination of CAR T-cells.

The original research article by Koski et al. focuses on modifying the spacer region between the antigen binding scFv and the transmembrane part of a CAR. Usually, IgG1-CH2-CH3 constant domains are used as spacers. However, these bare the risk of unwanted binding of bystander cells *in vivo*, such as monocytes/macrophages or NK cells, expressing Fc-receptors (FcR), which can result in unwanted myeloid cell activation and inflammation (IL-1β production), CAR T-cell activation (IL-2 and IFNγ production), and overall reduction of CAR T-cell activity towards the tumor cells. The novel SIRPα-based spacers developed by Koski et al. resulted in CARs that evaded the off-target binding to FcR without compromising the functionality of the CAR T-cells.

## 4 Conclusion

Although highly effective against hematologic neoplasms, CAR T-cell therapy is still less effective when applied to solid tumors. This is due to technical difficulties in CAR manufacturing, as well as biological mechanisms dampening CAR T-cell effectiveness. The evidence proposed and discussed in the studies collected in this Research Topic contributes to overcoming these issues and optimizing CAR T-cell usage in solid malignancies.

